# Festschrift in honor of Dr. Robert Stickgold, pioneer in sleep, memory, and dream research

**DOI:** 10.1093/sleepadvances/zpag041

**Published:** 2026-04-09

**Authors:** Tony J Cunningham, Erin J Wamsley, Jessica D Payne

**Affiliations:** Center for Sleep and Cognition, Department of Psychiatry, Beth Israel Deaconess Medical School, Boston, MA, 02215, United States; Division of Sleep Medicine, Harvard Medical School, Boston, 02215, MA, United States; Department of Psychology and Program in Neuroscience, Furman University, Greenville, SC, 29613, United States; Department of Psychology, University of Notre Dame, Notre Dame, IN, 46556, United States


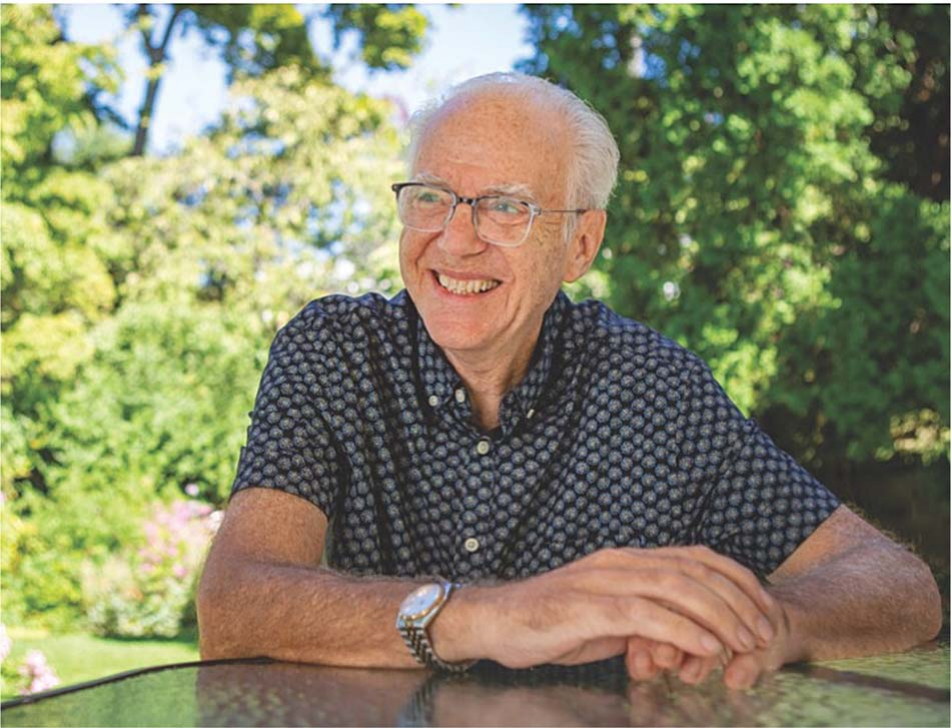


For the last few decades, Dr. Robert “Bob” Stickgold has led the way in reshaping our understanding of sleep through his influential studies and theoretical frameworks. His work has established sleep as an active component of memory consolidation, in which reactivation and dreaming foster the selective stabilization, abstraction, and integration of waking experiences, optimizing our cognitive and emotional functioning. His elegant experimental designs and theoretical intuition have moved the field forward methodologically and conceptually, and his lab at Harvard Medical School and Beth Israel Deaconess Medical Center (BIDMC) has been an intellectual home for generations of students and collaborators. This Festschrift (volume of peer-reviewed work from past trainees and colleagues) acknowledges his specific empirical contributions that established core paradigms in sleep-related learning, the broader intellectual position he has advocated—treating dreams and sleep physiology alike as legitimate, interpretable data that can be used to inform how the brain processes and remembers experiences—and the collegial, curious, and collaborative spirit in which he has pursued this line of work.

One of the most remarkable aspects of Bob’s trajectory and influence on the field is that sleep is his second scientific pursuit. After majoring in Biochemistry at Harvard as an undergraduate (Figure 1), he went on to earn his PhD in the same area from the University of Wisconsin. He then charted his early research path in that field as an Assistant Professor of Physiology at UMass Medical School [1–3] before transitioning to the private sector as an educational software designer for nearly a decade. To our good fortune, he returned to academia in the 1990’s and set his sights on the study of sleep, dreams, and cognition. His foundation in Biochemistry and medicine did more than just provide him with fodder for his short stories and science fiction novels (in true Renaissance Man fashion; see examples of his work here). It also provided him with a deep command of the molecular and cellular underpinnings of brain physiology, a perspective that enriched his work throughout his career. At the same time, his shift into cognitive neuroscience revealed a rare talent: the ability to hold the fine-grained details of neurochemistry and neuroanatomy in view while also stepping back to discern broader patterns, connections, and theoretical frameworks. It is this exceptional ability to understand the full spectrum of sleep medicine—from neural circuitry to human behavior—that has made his contributions so distinctive and impactful.

**Figure 1:**
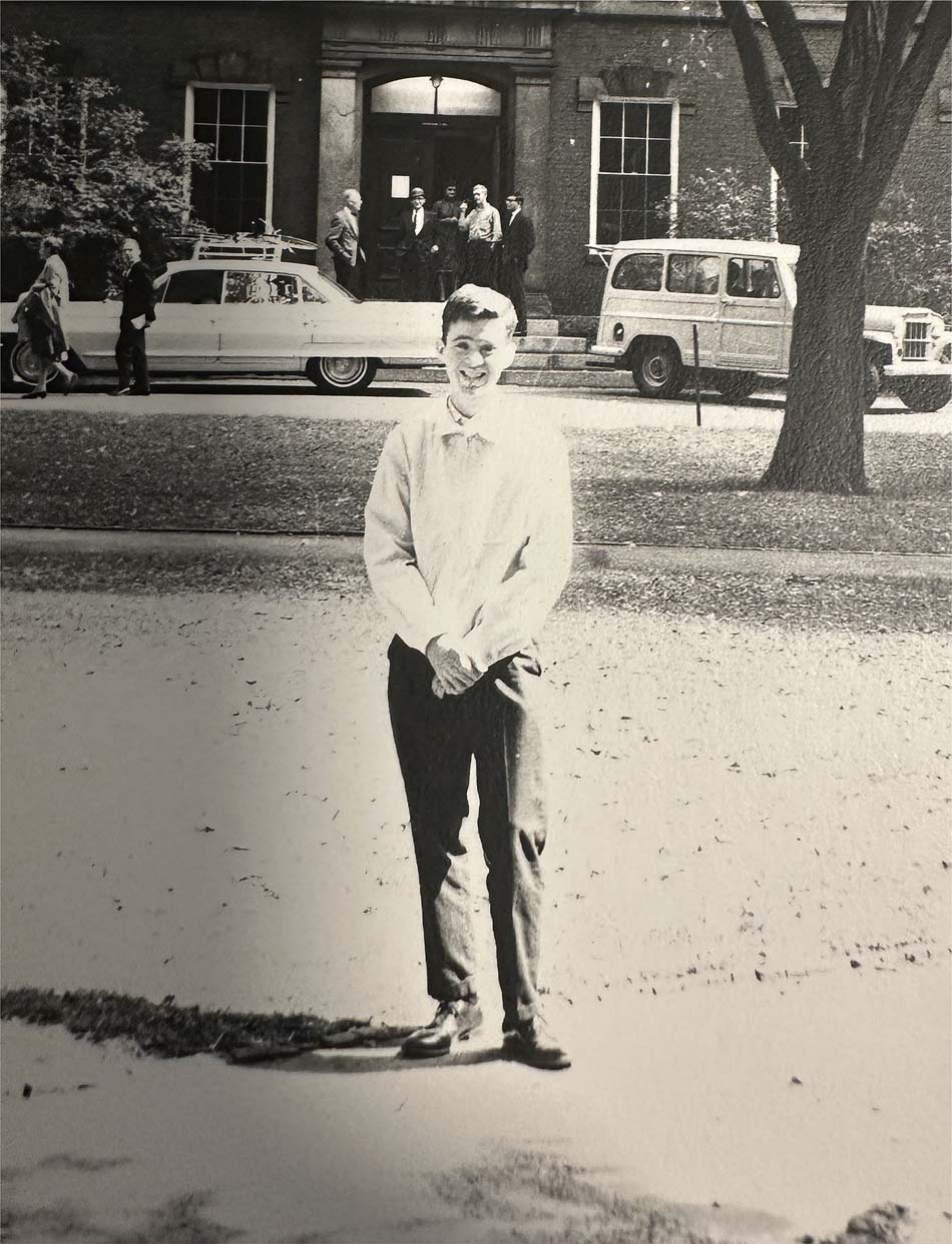
Pictured is a young Bob Stickgold launching his first academic journey at Harvard as an undergraduate. There he studied biochemistry and quickly developed a passion for scientific inquiry that would guide the rest of his career. Photo credit: Bob Stickgold, PhD

Bob’s return to academia brought him alongside J. Allan Hobson, a giant in the field of sleep and dreaming research [4–7]. Together, they began to fundamentally reshape how we understand sleep, transforming it from a passive downtime state into an active player in memory processing. This collaboration led to landmark publications as Bob and his team applied innovative cognitive tasks to traditional sleep and wake study designs. Their goal was to understand sleep’s role in cognition. Early breakthrough studies demonstrated that offline improvement on certain tasks, such as visual discrimination and procedural memory, occurs only after a period of sleep [8–10]. The field gained significant momentum when Bob’s work linked memory and cognitive gains to specific sleep features like Stage 2 sleep and sleep spindles [11–13]. From this foundation, the field grew exponentially (see Figure 2). Indeed, it was Bob’s pioneering work on sleep and cognition that inspired co-author Jess Payne to abandon her postdoctoral fellowship in stress research and join the Stickgold lab instead. Together, they explored sleep’s impact on explicit, episodic memories [14–16]. This exploration quickly revealed that sleep goes beyond simple memory stabilization to extract the gist of experiences and transform memories into useful guideposts for the future rather than mere replays of the past [13, 15, 17]. Bob’s mentorship tree grew a late branch when Jess convinced him to take her graduate student, Tony Cunningham, as “one final postdoc” before retirement. Together, Bob and Tony explored the cyclical relationship between sleep and emotional processing, investigating both the impact of sleep loss on emotional perception and memory [18–20], and the impact of socio-political stress on sleep during these turbulent times [21]. And now, driven by Bob’s endorsement and ceaseless support, the legacy of his Center for Sleep and Cognition at BIDMC continues with Tony taking over as the new director in 2023.

**Figure 2:**
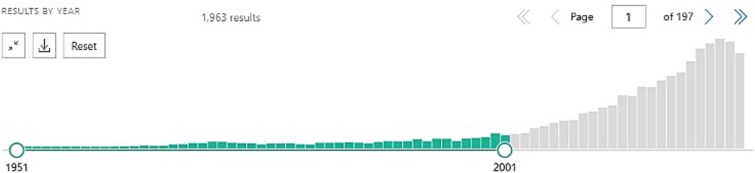
Bob’s early work on sleep and memory contributed to a subsequent tidal wave of research in the field, spawning interdisciplinary collaborations, funding opportunities, and even entire conferences dedicated to the topic. Image credit: Pubmed®

Guided by his leadership for more than two decades, Bob and his trainees have illuminated the critical importance of sleep oscillations and microarchitecture in the optimization of memory consolidation, transformation, and integration during sleep [14–17, 19, 22–26]. At regular intervals, Bob has produced high-profile reviews that demonstrate his remarkable ability to synthesize diverse research into integrative, forward-looking frameworks—a defining hallmark of Stickgold review papers [20, 27–34]. Through his writing, Bob has emphasized that sleep facilitates memory evolution by abstracting essential information, integrating experiences, and enabling us to generate creative new ideas based on past experience. Building on this basic science foundation, Bob’s recent work has focused on applying these findings to clinical populations [35–37], such as patients with schizophrenia [27, 38–41], and advocating for advanced methods to push the field forward.

While the search for precise mechanisms and boundary conditions continues [20, 42], it is now widely accepted that sleep represents an optimal period for memory processing and consolidation, a finding that has spurred interest in generations of subsequent research and academic inquiry. In this Festchrift alone, Barsky et al. report that reactivation during wakefulness fails to offer the performance improvement that sleep does [43]. Saletin, Carskadon, and colleagues provide evidence that sleep restriction impairs memory discrimination in an adolescent sample [44]. Likewise, Cunningham et al. found that total sleep deprivation severely impaired all forms of memory, but recovery sleep after total sleep deprivation not only preserves neutral memory and restores overall memory function to well-rested levels, but specifically enhances memory specifically for emotional content [45]. Work from Denis, Payne, and colleagues hint at the transformative potential of sleep, as sleep both strengthens and distorts story recollection [46]. More evidence is also offered on the importance sleep microarchitecture, as Mylonas, long-time collaborator Dr. Dara Manoach, and colleagues demonstrate that sleep fragmentation alone is not sufficient to account for spindle or memory deficits in individuals with chronic schizophrenia, but rather the deficit in the frequency of spindles is directly associated with impairment in memory consolidation [47]. Jourde et al. provide evidence that closed-loop auditory stimulation can reliably target ongoing sleep spindles without disrupting sleep, validating the method as a tool for future causal tests of the role of spindles in memory consolidation [48]. And finally, Shuster, Mednick, and colleagues offer a new theory on the role of REM sleep in memory consolidation as a mechanism designed to increase the signal-to-noise ratio within and across memory representations [49]. These contributions not only underscore the remarkable progress that has been made in unraveling the links between sleep and memory, but also highlight the exciting frontiers ahead, as novel methods and bold new theories promise to propel the field into its next era of discovery.

Concurrent with his work on sleep, Bob has led an impactful line of dream research contributing to some of the most significant advancements in our understanding of dreams in the last 50+ years [50–53]. Anyone who knows Bob knows that his research on dreaming is not a side project, but a driving passion. Over the last decades, his work has been instrumental in bringing rigorous cognitive neuroscience methodology to dream research, and in developing innovative new theory, enabling advances in an area where progress had stagnated for decades. One example of methodological innovation was the *Nightcap* at-home sleep staging device [54–56]. Long before the current explosion of at-home wearables, Bob developed this novel, low-cost device as a way to accurately detect sleep in the home environment. In contrast to older protocols requiring laborious manual all-night PSG monitoring in the laboratory, the *Nightcap* uniquely enabled numerous dream reports to be collected in quick succession across the full range of sleep stages, in participants’ home environment [57]. Among other discoveries, this approach facilitated Bob’s breakthrough *Science* paper demonstrating that amnesiac patients without a hippocampus dream about playing *Tetris* before sleep, even though they don’t remember playing it! [58] Those early studies with the *Nightcap* were a spark that first led guest editor Erin Wamsley to sleep research. In her high school library, she vividly recalls reading a *New York Times* article about the Nightcap and its creator “Mr. Stickgold,” on microfiche [59]. Years later, she and Stickgold together conducted some of the first studies testing whether dreaming about recent experiences affects later memory for them [60–62]. Together with work from other labs, this line of research established that the incorporation of learning experiences into dreaming is associated with enhanced memory consolation during sleep [63] and the *Tetris Effect* has gone on to enter the general lexicon, inspiring books, music, and video games (see Figure 3).

**Figure 3:**
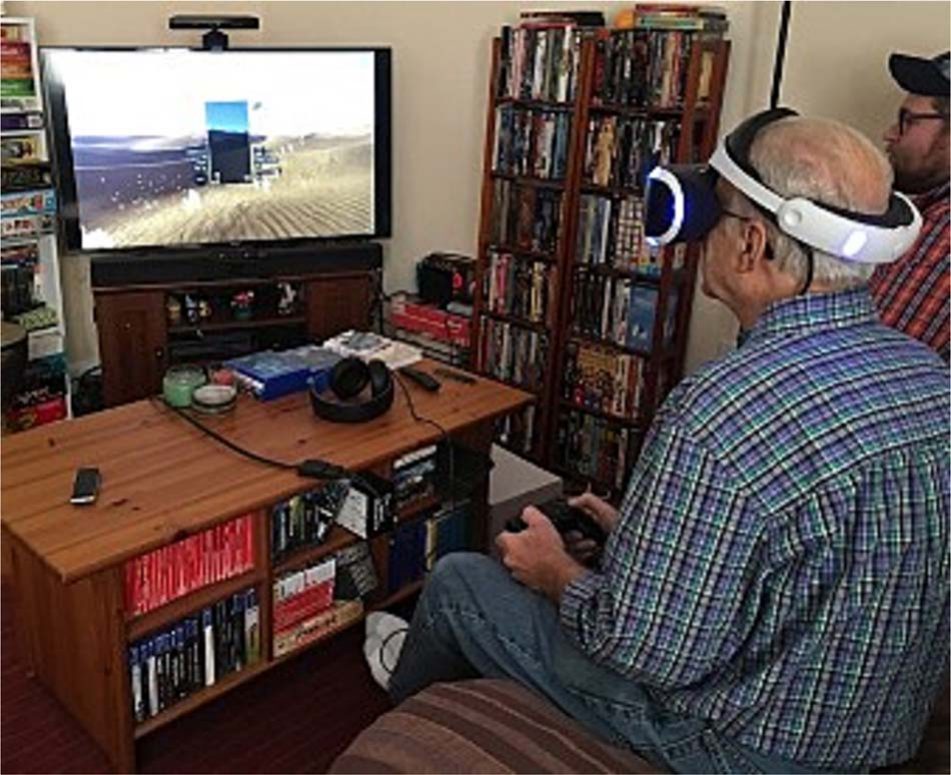
“Bob’s 2000 *Science* paper so influenced the public’s imagination that it spawned the popular term ‘The Tetris Effect’,” which later became the name of new versions of the classic *Tetris* video game. The award-winning *Tetris Effect* and *Tetris Effect: Connected* have been released on multiple gaming platforms, including those supported with virtual reality capabilities. Pictured is Bob taking the game inspired by his work for a test drive. Photo credit: Anna Schapiro, PhD.

Bob has continued his legacy of methodological innovation in recent years, for example collaborating with Adam Horowitz on research using the *Dormio* device to assist in *targeted dream incubation,* a technique for experimentally controlling dream content via pre-sleep suggestion [64, 65]. The *Dormio* detects sleep onset using innovative multimodal data including the progressive relaxation of hand muscles [64]. Using this device to automatically prompt mentation reports at the moment of sleep onset, Bob and Adam have shown that inducing dreams related to the topic of earlier creativity tasks enhances post-sleep creative performance [65]. This focus on how dreaming facilitates creativity is emblematic of Bob’s more recent theoretical direction, which moves beyond a simple focus on dreaming and memory, toward a more holistic view of dreaming as a process that the brain uses to find connections between past experiences, semantic memories, thought and feelings—ultimately enabling the creation of new meanings.

Bob’s NEXTUP model of dreaming (**N**etwork **Ex**ploration **tU**nderstand **P**ossibilities), developed together with Antonio Zadra, now formalizes his work on conscious experience during sleep into a comprehensive model of how the brain creates novel meaning during sleep [53]. This work is inspiring the next generation of dream researchers to focus on how sleep and dreaming not just “strengthen” memory, but use past experience in service of creating new ideas. Exemplifying this, in the current issue, Wamsley, Trost, and Tucker report on the combination of related memories in dreams, highly congruent with key predictions of NEXTUP [66]. Fechner, Born, and colleagues provide evidence that having intentions to complete a task the next morning impacts the content of the dream, making it more semantically related [67]. In work from the lab of Bob’s long-time colleague, Dr. Edward Pace-Schott, McGory et al. report that script rehearsals of trauma-related nightmares elicit the same degree of emotional response as the rehearsal of the index trauma itself [68]. Youngren and colleagues report that targeted dream incubation leads to increased self-efficacy during dreaming, which they hope to leverage to enhance sleep-related interventions, such as treatments for these trauma-related nightmares [69]. And Bob’s own submission calls the frequently sought thematic coherence of dream construction into question, suggesting different approaches are necessary to advance the field forward [70]. We look forward to seeing the further advances that this theory inspires in coming years!

The papers included as part of this special Festschrift provide a window into the far-reaching influence that Bob has had on the field. Across his career, Bob has contributed to over 250 scholarly articles, has been a PI or co-investigator on over 20 major grants, and was the recipient of the 2024 Harvard Medical School Division of Sleep Medicine Prize in recognition of his outstanding lifetime contributions to the field of sleep and circadian rhythms. But beyond his academic work, Bob has been a long-time advocate for the field of sleep, inspiring both the general public and funding organizations alike with his scores of interviews and writings for the lay community, including his 2021 book, *When Brains Dream* [53]. His career exemplifies how careful experimentation, theoretical courage, and intellectual collaboration can produce durable shifts in scientific perspective. This Festschrift recognizes not only the discoveries he made during his career, but the questions he generated for the field that follows in his wake.

## Notes

*This is a special collection article Festschrift in honor of Dr. Robert Stickgold*.
